# Multifunctional TPP-PEG-biotin self-assembled nanoparticle drug delivery-based combination therapeutic approach for co-targeting of GRP78 and lysosome

**DOI:** 10.1186/s12951-020-00661-y

**Published:** 2020-07-20

**Authors:** Baskaran Purushothaman, Jeongmin Lee, Sera Hong, Joon Myong Song

**Affiliations:** College of Pharmacy, Seoul National University, Seoul, 08826 South Korea

**Keywords:** TPP-PEG-biotin self-assembly, Combination therapy, Autophagy, GRP78, Co-targeting

## Abstract

**Background:**

In this study, a multifunctional tetraphenylporphyrin (TPP) conjugated polyethylene glycol with biotin (TPP-PEG-biotin) as a photo-dynamic therapy (PDT) material encapsulating a ruthenium complex 1 (Ru-1) was fabricated as self-assembled nanoparticle (Ru-1@TPP-PEG-biotin SAN) to co-target glucose-regulated protein 78 (GRP78) and the lysosome as a new anti-cancer therapeutic strategy.

**Results:**

The MTT assay results reveals the enhanced anticancer activity of the Ru-1@TPP-PEG-biotin SANs due to the co-targeting of the GRP78 and lysosome. The Ru-1@TPP-PEG-biotin reduced level of GRP78 and lysosomal ceramide that contributed to the stability of the lysosomal membrane. The endoplasmic reticulum (ER) stress concomitant with the inhibition of GRP78 was clearly monitored by the phosphorylation of protein kinase R (PKR)-like endoplasmic reticulum kinase (PERK), and inositol-requiring enzyme 1 α (IRE1α) kinases to indicate the activation of the unfolded protein response (UPR) signaling using immunofluorescence assay. On the other hand, the degradation of the lysosome was observed through PDT action by the Ru-1@TPP-PEG-biotin SAN treatment. This was confirmed by the co-localization assay showing the disappearance of cathepsin D and lysosomal-associated membrane protein 1 (LAMP1) in the lysosome.

**Conclusions:**

Considering lysosome-mediated autophagy is an effective cancer cell survival mechanism, the degradation of the lysosome along with GRP78 inhibition by the Ru-1@TPP-PEG-biotin SAN combination therapy is suggested as a new co-targeting cancer treatment.
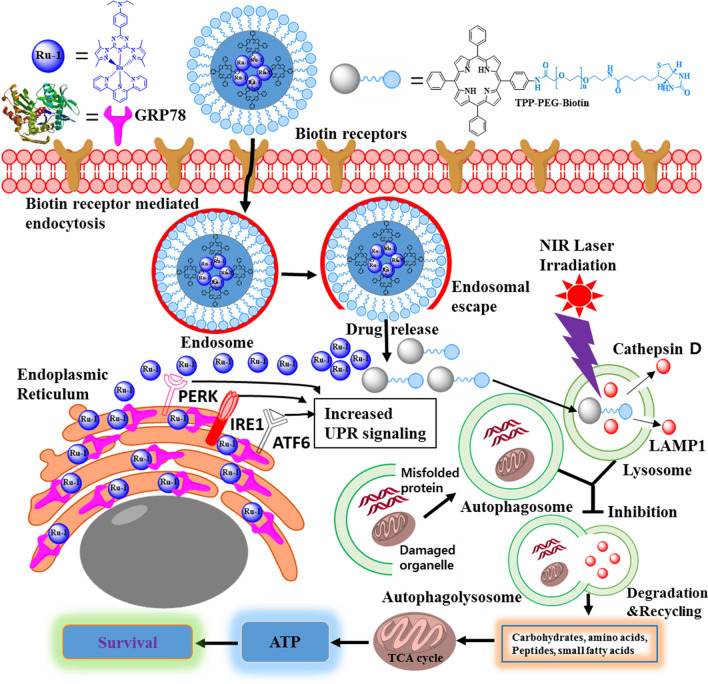

## Background

Chemotherapy is the most historic and basic anti-cancer drug therapy. However, most existing chemotherapy drugs show multi-drug resistance in many malignancy patients [1]. In addition, since chemo drugs cannot distinguish between cancer cells and normal cells, serious side effects often occur. New forms of innovation are needed to overcome multi-drug resistance and serious side effects and to enhance the therapeutic effect. Combination therapy has been proposed as an effective anti-cancer strategy to deal with multi-drug resistance and side effects [2]. The nanoparticle drug delivery system (NDDS)-mediated combination therapy is a widely used method of treating cancers with two or more different types of anti-cancer drugs at the same time [3, 4]. If only a single type of anti-cancer drug is used, the drug cannot be administered at a high concentration due to side effects. When the different action mechanisms of the anti-cancer drugs produce synergistic effects to each other, they exhibit strong anti-cancer effects at smaller doses, and it reduces the side effects and drug resistance. Although NDDS-mediated combination therapy of chemotherapeutic agents has been extensively explored, NDDS-based combination therapy is still needed to combat drug resistant tumors for better treatment based on finding new targets.

GRP78, a type of heat shock protein 70 (HSP70), has been reported to be one of the biomarkers overexpressed in cancer cells and cancer stem cells [5, 6]. GRP78 facilitates formation of the S–S bonds of proteins to produce proteins with normal stereoscopic structures. GRP78 has a role as a quality control manager that prevents accumulation of unfolded proteins and produces structurally active proteins in the endoplasmic reticulum (ER). As a result, the efficiency of the intracellular energy consumption can be maintained well. In the tumor microenvironment, GRP78 causes cancer cells to adapt to chronic stress and promotes the proliferation, survival, metastasis and resistance to drugs [6]. Lysosome affects the inactivation of damaged intracellular organelles, the downward regulation of cell receptors, and the cell membrane reconstruction and rotation rate of cellular components through in-cell cleaning activities [7]. The lysosome digests dysfunctional subcellular organelles and materials in the process of autophagy and enables the reuse of basic components such as proteins, glycosaminoglycans, glycogen, nucleic acids, etc. [8]. When dysfunctional macromolecules are not digested and accumulate in the cells, metabolic energy efficiency is reduced, and in severe cases, lysosomal storage disorders may occur. Cancer cells need many functional proteins because of their faster cell division compared to normal cells. Therefore, molecular chaperones like GRP78 are overexpressed so that the ER is not overloaded. When both GRP78 and lysosome are destroyed by co-targeting therapy, the by-products including unfolded proteins due to GRP78 inhibition are not be cleaned by the lysosome. These by-products accumulate in the cancer cells, and the supply of basic unit materials for new protein synthesis is not readily available. Therefore, co-targeting both GRP78 and lysosome could be newly suggested to be an attractive anti-cancer therapeutic strategy that provides cancer cells with the worst energy efficiency, based on the mass production of unfolded proteins which cannot be digested.

In addition, ruthenium(III, II) complexes have shown strong affinities towards thiol containing proteins such as bovine serum albumin (BSA), glutathione (GSH), and transferrin [9]. Particularly, their strong affinities to transferrin is known to cause more accumulation in cancer cells than in normal cells due to an active metabolism that needs more Fe^2+^ ion [10, 11]. TPP-PEG-biotin is a PDT substance that produces reactive oxygen species (ROSs) by irradiation at 660 nm [12]. Due to the covalent bonding of TPP to PEG, this material can act as self-assembly-based NDDS and a photosensitizer. The biotin moiety in TPP-PEG-biotin can be effectively delivered to cancer cells, such as MCF-7 and HepG2, in which biotin receptors are overexpressed. In addition, TPP-PEG-biotin was reported to be localized in the lysosome of MCF-7 cells [12]. The self-assembled nanoparticles (SANs) of TPP-PEG-biotin can encapsulate chemo drugs targeting different subcellular organelles. The molecular self-assembly is characterized as a process of making structurally well-defined aggregates through non-covalent interactions, including electrostatic, π–π, and hydrophobic interactions. Recently, SANs for NDDS have received much attention due to their significant advantages such as high drug loading, high hydrophilicity in nature, and low cytotoxicity [3, 13, 14]. In this study, Ru-1 loaded TPP-PEG-biotin self-assembled nanoparticles (Ru-1@TPP-PEG-biotin) was used in a novel combination therapy that simultaneously co-targeted GRP78 and lysosome as a new anti-cancer therapeutic strategy. Compared to previous approach based on encapsulation of doxorubicin using TPP-PEG-biotin NDDS [12], this study deals with a new co-target. Efficacy of combination therapy can be variable as a function of drug target. Different targets by therapeutic agents provide cancer cells with different damages which may be more lethal to them. The co-targeting of GRP78 and lysosome is expected to be a very efficient anti-cancer therapeutic strategy in that the worst energy efficiency is given to cancer cells. In the previous work [11], Ru-1 has been identified as a potent anticancer agent against drug resistant cancer stem cells (CSCs). The hydrophobic property inherent to Ru-1 requires assistance of NDDS for active use of Ru-1 in a wide range of biomedical applications. Successful drug delivery of the synthetic Ru-1 is demonstrated for a new co-targeting using TPP-PEG-biotin SAN.

## Results

### Synthesis and characterization of Ru-1@TPP-PEG-biotin SANs

**Scheme 1:**
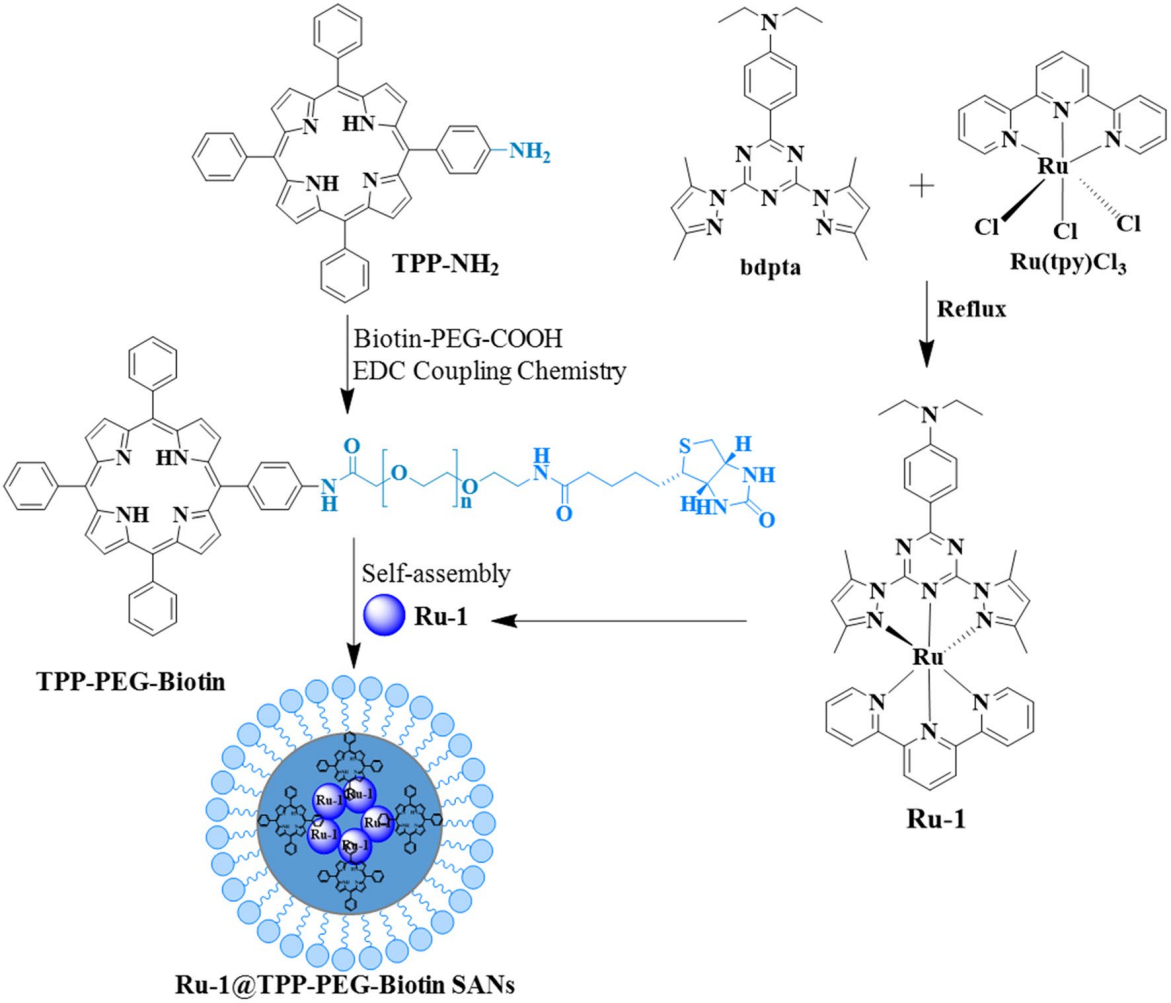
Synthetic route for the preparation of TPP-PEG-biotin SANs encapsulating Ru-1

**Fig. 1:**
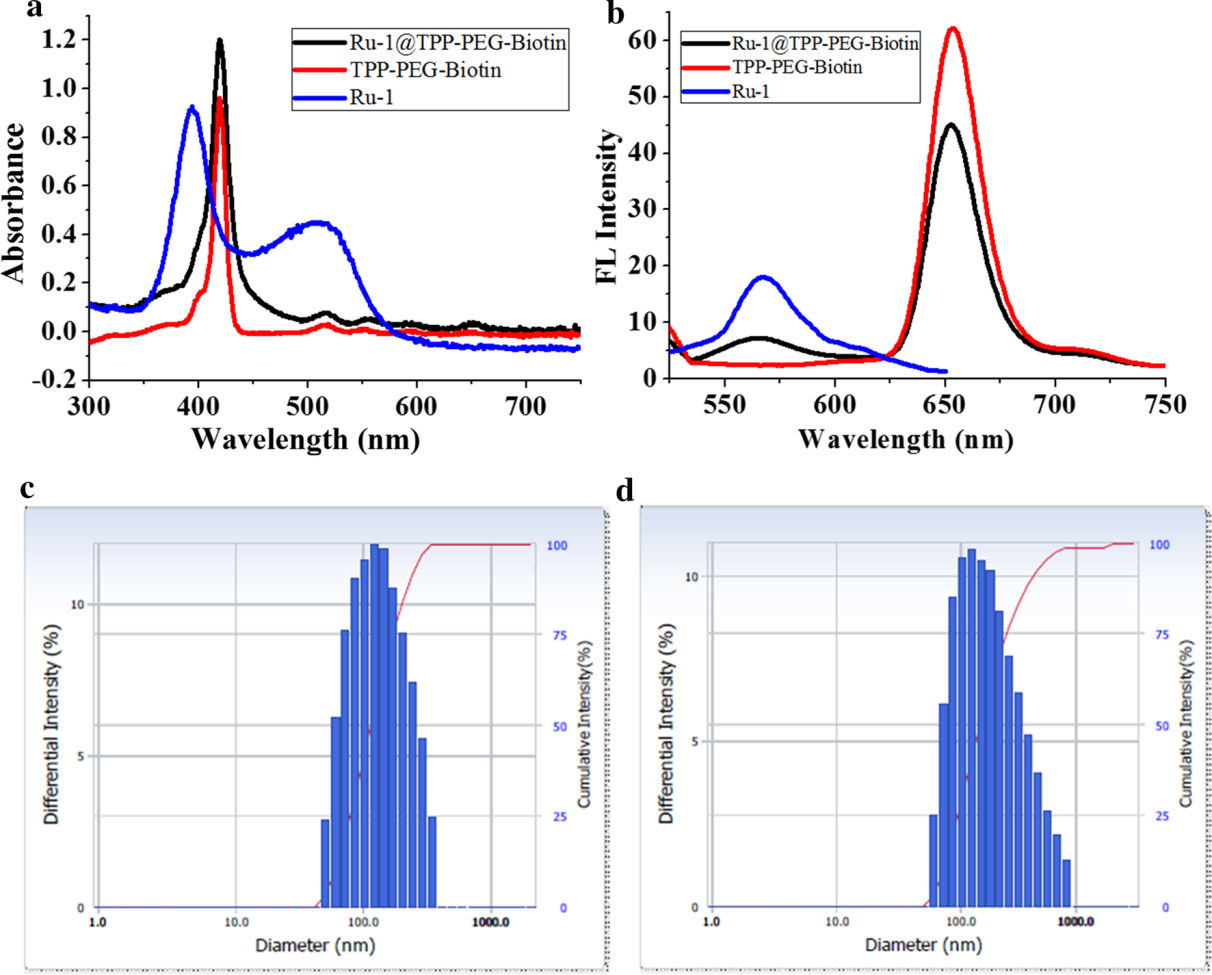
**a** UV–visible absorption and **b** emission spectra of Ru-1, TPP-PEG-biotin SANs, and Ru-1@TPP-PEG-biotin SANs. Particle size distribution of **c** TPP-PEG-biotin SANs and **d** Ru-1@TPP-PEG-biotin SANs based on dynamic light scattering

**Fig. 2:**
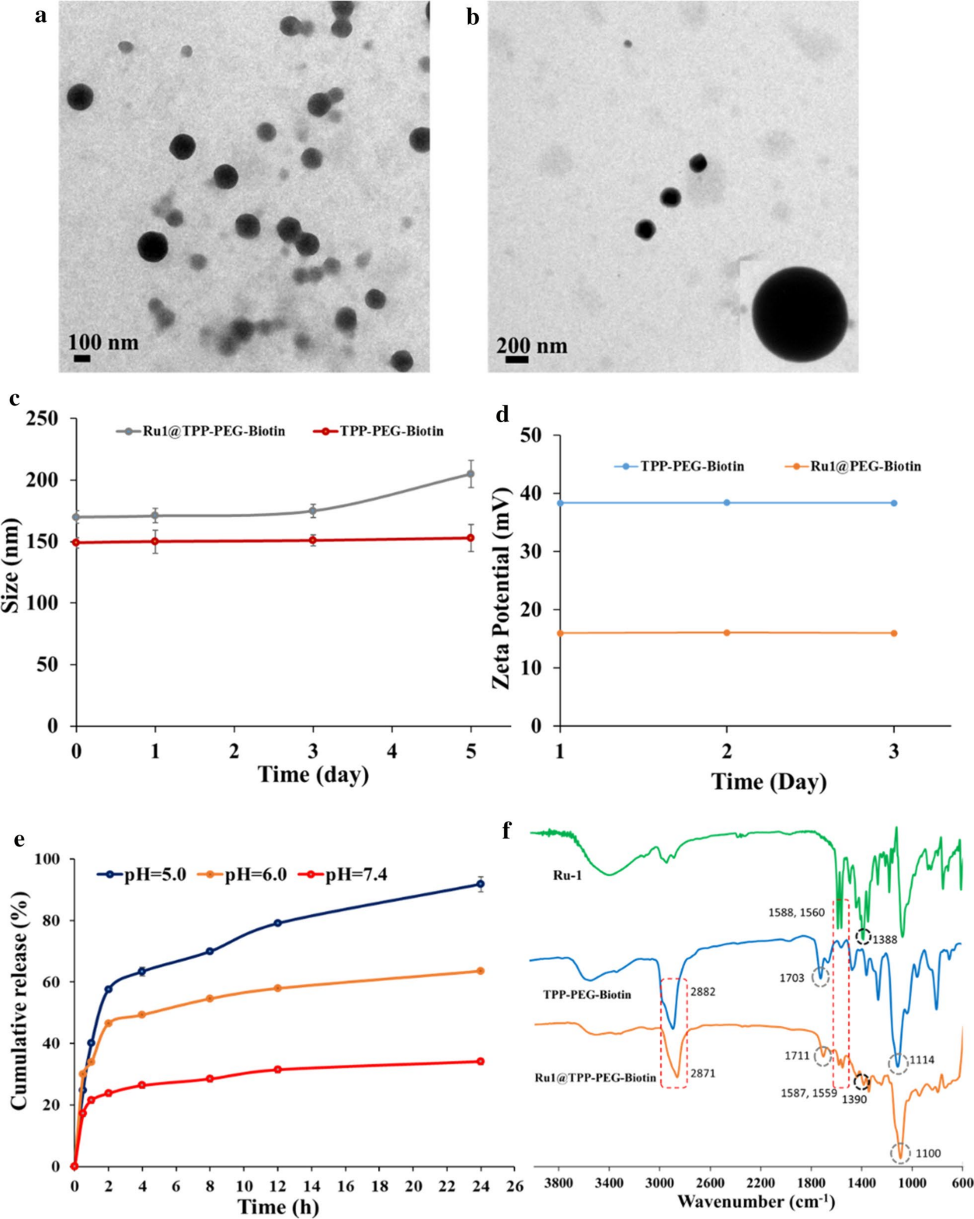
**a**, **b** The TEM images of the Ru-1@TPP-PEG-biotin SAN. In vitro **c** size and **d** charge stability measurement of TPP-PEG-biotin SANs and DOX@TPP-PEG-biotin SANs with PBS (pH 7.4). **e** In vitro Ru-1 release from the Ru-1 loaded TPP-PEG-biotin SANs at 37 °C under different PBS buffer (pH 7.4, pH 6.0 and pH 5.0) conditions. **f** The FT-IR spectra of Ru-1 (green), TPP-PEG-biotin SAN (blue), and Ru-1@TPP-PEG-biotin SAN (orange)

**Scheme 2:**
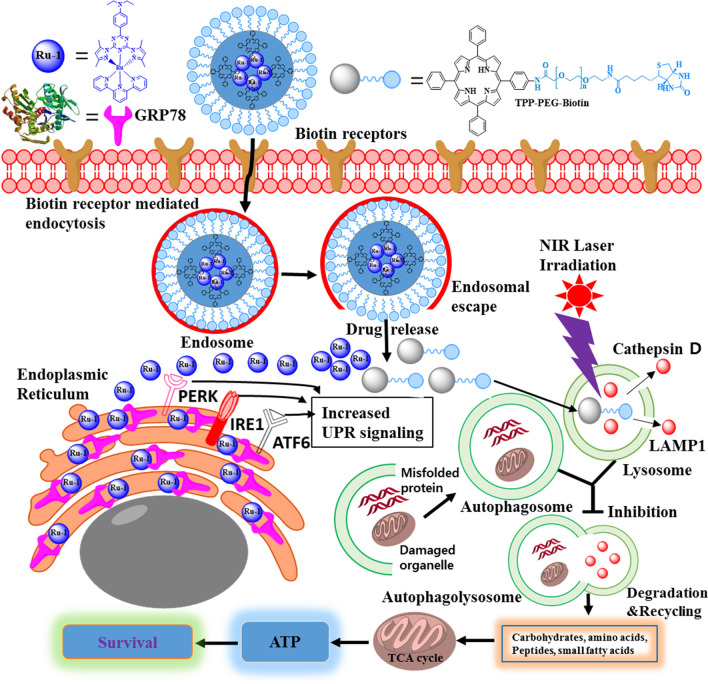
Co-targeting principle of GRP78 and lysosome by the TPP-PEG-biotin SANs encapsulating Ru-1

The synthetic route for the Ru-1 loaded TPP-PEG-biotin SANs is shown in Scheme 1. By using the Ru-1 and targeting moiety-functionalized TPP-PEG-biotin, a NDDS-based chemo-photodynamic combination agent was designed and prepared through sonication-assisted self-assembly [15, 16]. The Ru-1, TPP-PEG-biotin SAN, and Ru-1 loaded TPP-PEG-biotin SANs were analyzed by UV–Vis absorption and emission spectroscopy. The UV–Vis spectrum of TPP-PEG-Biotin SANs showed the Soret band at 418 nm and Q-band at 518 nm. On the other hand, the Ru-1@TPP-PEG-biotin SAN represented the Soret band at 420 nm and Q-band at 520 nm in the absorption spectrum (Fig. 1a). The emission spectrum of the Ru-1@TPP-PEG-biotin showed a peak at 566 nm corresponding to the Ru-1 and another peak at 654 nm. This spectral pattern is different from that of TPP-PEG-biotin alone with only an emission peak at 649 nm under the same excitation at 420 nm (Fig. 1b). The size of the Ru-1@TPP-PEG-biotin SANs was measured by dynamic light scattering (DLS). The mean hydrodynamic diameter of the Ru-1@TPP-PEG-biotin SANs was found to be 174 nm (Fig. 1d). On the other hand, the mean hydrodynamic diameter of the TPP-PEG-biotin SANs alone was measured to be 150 nm (Fig. 1c). The morphology of Ru-1@TPP-PEG-biotin SANs was measured using a TEM and their spherical shape was confirmed (Fig. 2a and b). The TEM results represented that after Ru-1 loading the average size of the particles was slightly increased (175 ± 30 nm), compared to TPP-PEG-biotin SANs alone (150 ± 25 nm) [12]. Further, the Ru-1 loading into the TPP-PEG-biotin SANs was analyzed by using FT-IR spectroscopy. The FT-IR spectra of the Ru-1 (green), TPP-PEG-biotin SANs (blue), and Ru-1@TPP-PEG-Biotin SANs (Orange) were shown in Fig. 2f. The broad peaks around 2880 and 1100 cm^−1^ correspond to the –CH_2_ and C–O–C stretching of PEG molecules, which have been observed in both TPP-PEG-biotin SANs and Ru-1 loaded TPP-PEG-biotin SANs. The Ru-1 contains the aromatic rings, ethyl, and methyl functional moieties. The asymmetric and symmetric bending frequency of –CH_3_ have been identified at 1490 and 1351 cm^−1^, respectively. The –NH–CO–NH-functional group in biotin moiety have showed the C=O stretching at 1703 cm^−1^. The Ru-1 loaded TPP-PEG-biotin SANs also showed the C=O stretching at 1711 cm^−1^. The symmetric aliphatic C–H bending of –CH_3_ group in Ru-1 observed at 1388 cm^−1^. The –CH_3_ symmetric stretching was also observed in Ru-1 loaded TPP-PEG-biotin SANs at 1390 cm^−1^. The strong C=C and C–C stretching of aromatic rings of Ru-1 complex have showed two base peak at 1588 and 1560 cm^−1^ respectively. These two peaks of C=C and C–C stretching of aromatic rings in Ru-1 were also observed in Ru-1@TPP-PEG-biotin SANs at 1587 and 1559 cm^−1^. The successful loading of Ru-1 into the TPP-PEG-biotin SANs was confirmed by stretching frequencies in FT-IR spectrum. These results show the formation of Ru-1 encapsulating SANs by the hydrophobic and π–π interactions between the TPP-PEG-biotin SANs and Ru-1. The schematic illustration of drug release and their co-targeting mechanism is shown in Scheme 2.

### In vitro drug release and stability of nanoparticles

The in vitro release profile of Ru-1 from the Ru-1@TPP-PEG-biotin SANs was evaluated by dialysis method at different pH values (pH 5.0, pH 6.0, and pH 7.4). Figure 2e shows the release profile of Ru-1 from the TPP-PEG-biotin SANs at 37 °C in the different PBS media (pH 5.0, pH 6.0, and pH 7.4). The drug release profiles show the sustained release of the Ru-1 from the SANs. However, the release rates of Ru-1 at acidic conditions (pH 5.0 and pH 6.0) were faster compared to the neutral condition (pH 7.4). The Ru-1 may have high affinity to TPP-PEG-biotin during the self-assembly process due to the hydrophobic and π–π stacking interactions [17]. An interesting feature of π–π stacking interactions is that the interaction forces are affected by environmental conditions such as pH and redox potential [18–21]. In addition, the π–π stacking interactions is very weak compared to the covalent bonding. The pH is a major parameter that affects the release of the π–π stacked drug from the drug loaded self-assembled nanoparticle system [18]. The Ru-1 in Ru-1@TPP-PEG-biotin SANs is thought to stably bind to TPP via the hydrophobic and π–π interaction under the neutral pH condition. However, the binding of Ru-1 to TPP in Ru-1@TPP-PEG-biotin SANs can be weak under acidic conditions as result of protonation of Ru-1. The Ru-1 has the tertiary amine functional group and triazine ring with free nitrogen lone pair electrons. These functional moieties can be protonated easily under acidic conditions. The protonated tertiary amine and triazine ring may hamper the interactions between Ru-1 and TPP in Ru-1@TPP-PEG-biotin SANs. Hence, under acidic pH conditions, Ru-1 can be released more from Ru-1@TPP-PEG-biotin SANs, compared to the neutral pH. As a result, the larger amount of Ru-1 from Ru-1@TPP-PEG-biotin SANs was observed under the acidic conditions, compared to neutral pH, as shown in Fig. 2e. After 24 h, the Ru-1 was released 34% at pH 7.4. On the other hand, under the acidic conditions, the Ru-1 releases of 63% and 91% were observed at pH 6.0 and pH 5.0, respectively. These results indicate that the encapsulated Ru-1 can be well released into the cancer cells from the TPP-PEG-biotin SANs.

The size stability of TPP-PEG-biotin and Ru-1@TPP-PEG-biotin SANs was analyzed using DLS. As shown in Fig. 2c, the SANs did not show any aggregation in the PBS at room temperature for five days (Fig. 2c). The DLS results indicate that the SANs prepared in this study possess an adequate stability. The zeta potential before and after the encapsulation of Ru-1 into SANs was measured using ELS to investigate the charge stability of the SANs (Fig. 2d). The zeta potential of TPP-PEG-biotin SANs alone was found to be +38.4 mV while that of Ru-1@TPP-PEG-biotin SANs was found to be +16.0 mV. After the encapsulation of Ru-1, the zeta potential of the Ru-1-loaded TPP-PEG-biotin SANs was reduced. The reduced zeta potential of Ru-1-loaded TPP-PEG-biotin SANs may be due to the adsorption of Ru-1 into the surface of TPP-PEG-biotin SANs. The loaded Ru-1 molecules may shift the plane of shear to a longer distance from the surface of SANs. This can lead to a reduction of the measured zeta potential [22–24]. This signifies even in the case of highly charged particle surface, a moderately low zeta potential can be measured. Although the smaller zeta potential of the Ru-1@TPP-PEG-biotin SANs was measured, the SANs were found to be stable in buffer solution. In this study, both TPP-PEG-biotin SANs and Ru-1-loaded TPP-PEG-biotin SANs have showed the positively charged surface. The positively charged SANs may preferentially be taken up by cancer cells because of the negatively charged phosphatidylserine residue on the cell surface.

### Cytotoxicity tests

**Fig. 3:**
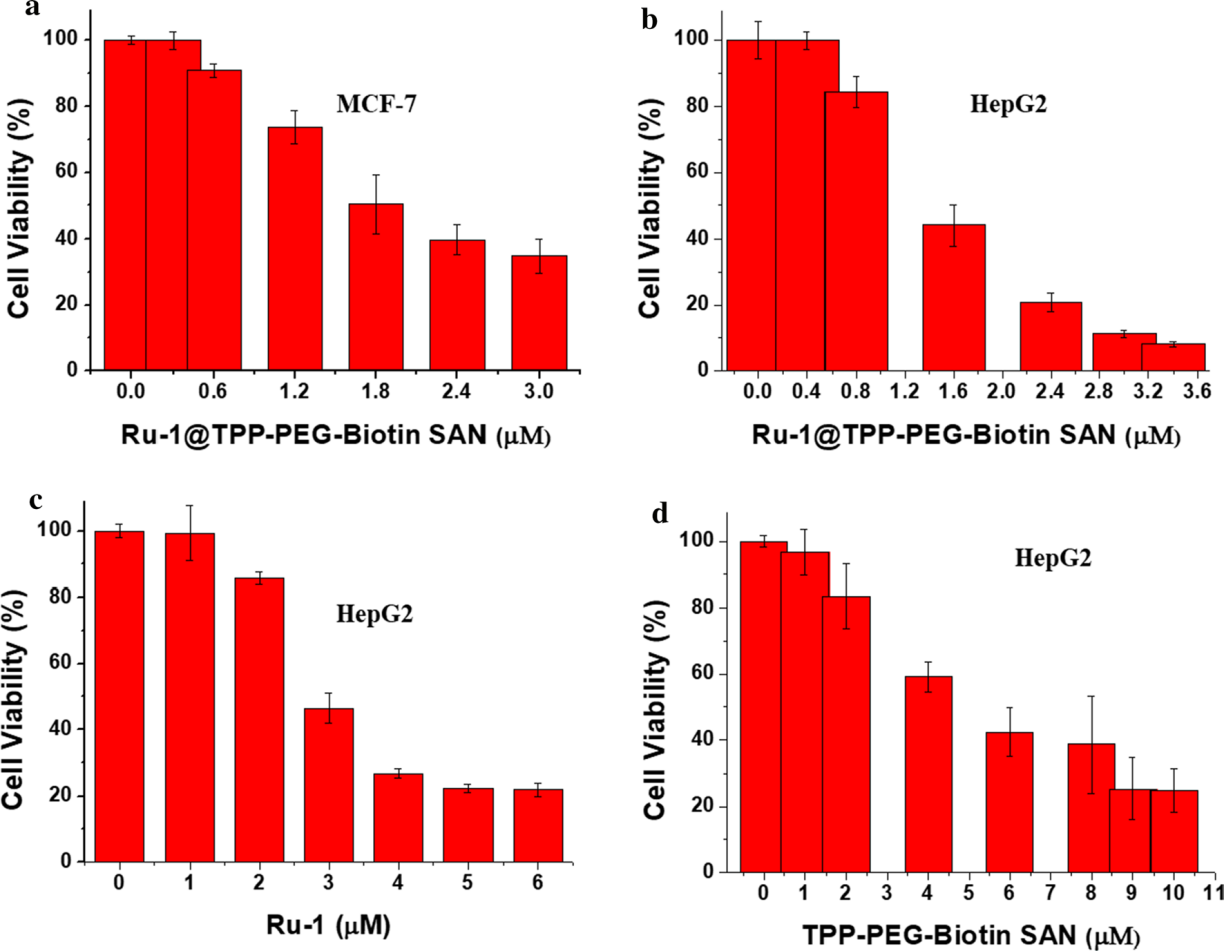
Cytotoxicity test. The cell viability of the Ru-1, TPP-PEG-biotin, and Ru-1@TPP-PEG-biotin SAN show negligible cytotoxicity. The cell viability of Ru-1@TPP-PEG-biotin SAN in **a** MCF-7 cells and **b** HepG2 cells under the light condition. **c** The cell viability of Ru-1 in HepG2 cells under dark condition, **d** The cell viability of TPP-PEG-biotin in HepG2 cells under light condition. Light dose: 660 nm, 30 mW/cm^2^, 20 min. The data were expressed as Mean ± SD, n = 3 independent experiments

Figure 3 shows the results of the MTT assays executed in HepG2 at different concentrations of Ru-1, TPP-PEG-biotin SANs, and Ru-1@TPP-PEG-biotin SANs. In the case of the MCF-7 cell line, only the cytotoxicity by the Ru-1@TPP-PEG-biotin SANs was measured under PDT conditions. The IC_50_ values by the individual Ru-1 and TPP-PEG-biotin SANs in the MCF-7 cell line were referred to the previous results [11, 12]. As shown in Fig. 3, HepG2 cell viabilities decreased linearly as the concentrations of Ru-1, TPP-PEG-biotin SANs, and Ru-1@TPP-PEG-biotin SANs increased. Based on the linear range, the IC_50_ values were obtained. Additionally, the MCF-7 cell viability showed a similar trend with respect to the Ru-1@TPP-PEG-biotin SANs. The calculated IC_50_ values of Ru-1, TPP-PEG-biotin SANs, and Ru-1@TPP-PEG-biotin SANs are presented in Table 1. In the HepG2 cell line, the IC_50_ value of the Ru-1@TPP-PEG-biotin SANs was determined to be 1.55 µM. This value is smaller than the IC_50_ values of the individual Ru-1 and TPP-PEG-biotin SANs. This result reveals the enhanced anticancer activity of the Ru-1@TPP-PEG-biotin SANs combination therapy due to the co-targeting of the ER and lysosome. Also, as shown by the IC_50_ value of 1.84 µM, the Ru-1@TPP-PEG-biotin SANs represented an increased anticancer activity in the MCF-7 cell line due to the combination therapy. The IC_50_ value of DOX@TPP-PEG-biotin SANs was found to be 1.05 µM against MCF-7 cells in previous work [12] and showed similarity to that of Ru-1@TPP-PEG-biotin SANs (1.84 µM), particularly considering IC_50_ value (1.56 µM) of DOX alone and that of Ru-1 alone (2.1 µM) against MCF-cells.

**Table 1:** IC_50_ values for Ru-1, TPP-PEG-biotin SAN, and Ru-1@TPP-PEG-biotin SANs against MCF-7 and HepG2 cancer cells

		IC_50_ values (µM)	
Cell lines	Ru-1	TPP-PEG-biotin SAN	Ru-1@TPP-PEG-biotin SAN
MCF-7	2.10*	4.74*	1.84
HepG2	3.01	5.92	1.55

Cell viability was determined by MTT assay after 24 h incubation (mean of three independent experiments ± SD). * reported values [11, 12]

The IC_50_ values of TPP-PEG-biotin SAN, and Ru-1@TPP-PEG-biotin SANs were acquired based upon MTT assay results under the light irradiation. The IC_50_ value of Ru-1 was obtained under the dark condition. Light dose: 660 nm, 30 mW/cm^2^, 20 min

### Intracellular drug release

The intracellular Ru-1 drug release and delivery behavior of Ru-1@TPP-PEG-biotin SANs was assessed using confocal laser fluorescence microscopy (CLFM) images (Additional file 1: Fig. S1a). MCF-7 cells were incubated with free Ru-1 and Ru-1@TPP-PEG-biotin SANs at 37 °C, and then the Ru-1 release profiles of Ru-1@TPP-PEG-biotin SANs were evaluated at different time intervals (3 h, 12 h and 24 h) by CLFM image analysis. Additional file 1: Fig. S1b presents the quantitative analysis for the fluorescence intensity of Ru-1 release. The CLFM image results of Ru-1@TPP-PEG-biotin SANs showed that fluorescence intensity of Ru-1 became higher with increasing incubation time. In addition, Ru-1 fluorescence in Ru-1@TPP-PEG-biotin SANs was observed more strongly than in the free Ru-1, indicating that the free Ru-1 was less distributed and delivered in MCF-7 cells compared with the Ru-1 loaded into TPP-PEG-biotin self-assembled nanoparticles. This result demonstrated that the conjugated biotin moiety of TPP-PEG-biotin SANs contributes to preferential uptake by the MCF-7 that overexpress the biotin receptor. Moreover, the Ru-1 was efficiently released from the Ru-1@TPP-PEG-biotin SANs in MCF-7 cells.

### Subcellular localization (glucose-regulated protein)

**Fig. 4:**
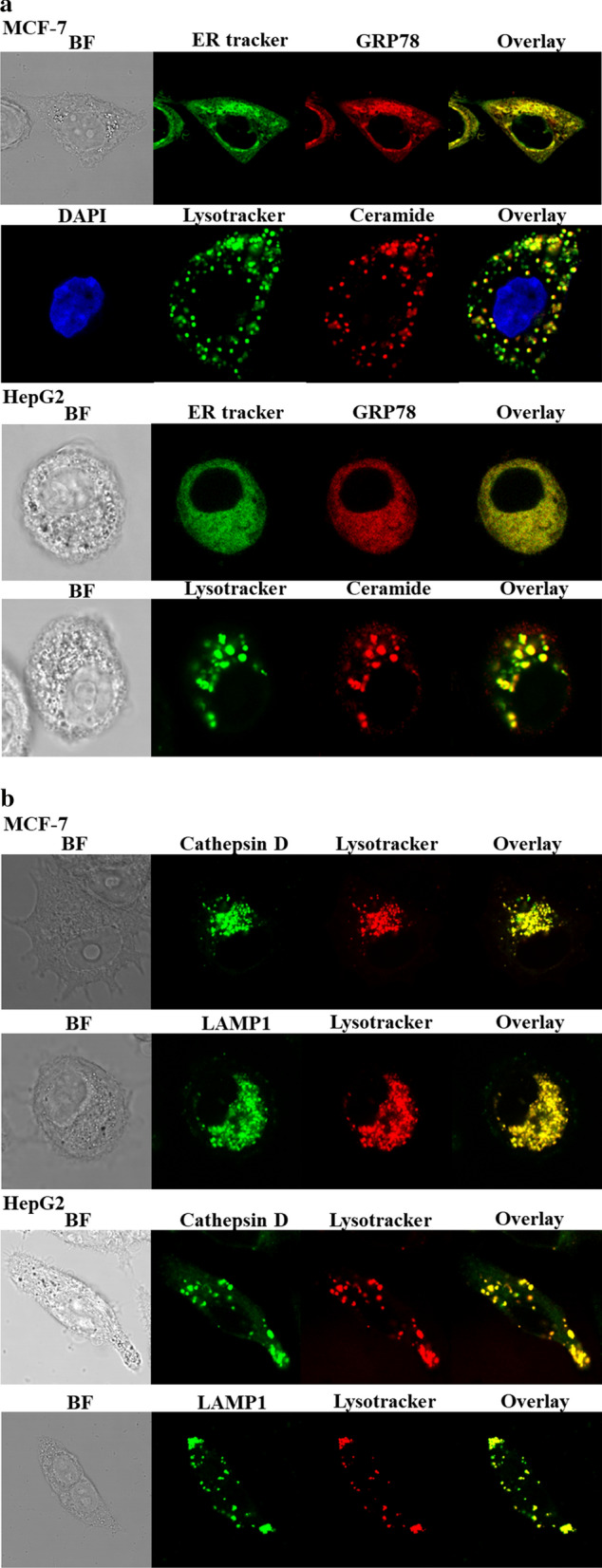
Subcellular localization images of GRP78, ceramide, cathepsin D, and LAMP1 in MCF-7 and HepG2 cells using confocal fluorescence microscopy. **a** The subcellular localizations of GRP78 and ceramide were observed by ER tracker and lysotracker, respectively. **b** The subcellular localization of cathepsin D and LAMP1 in the lysosome was monitored using lysotracker. ER tracker: 504/511(Ex/Em), Lysotracker: 577/590 (Ex/Em)

GRP78 interacts with bis(monoacylglycerol) phosphate (BMP), an anionic lipid phosphate bound to the inner lysosomal membrane, which is involved in the generation of ceramide in the lysosome. Ceramide is known to contribute to the membrane stabilization of lysosome [25]. GRP78 exists mainly in the endoplasmic reticulum (ER) and in the cytosol. Intracellular localization of GRP78 was investigated by fluorescence monitoring of the ER tracker and GRP78 antibody labeled with a fluorophore. Subcellular organelle localization of ceramide in the lysosome was also observed using the lysotracker and ceramide antibody labeled with a fluorophore. Figure 4a shows GRP78 is localized mainly in the ER and partially in the cytosol in the MCF-7 and HepG2 cell lines. As a result of the localization of most of the GRP78 proteins in the ER, strong yellow spots are observed by the overlap of the GRP78 red and ER green fluorescence images. Partially red spots in the overlapped image reveal GRP78 proteins exist in the cytosol. In the case of ceramide, the overlapped image indicates that almost all the ceramide is localized in the lysosome of the MCF-7 and HepG2 cell lines. Cathepsin D is one of the enzymes that are mainly present in the lysosome, and LAMP1 is one of the lysosome-associated membrane proteins. Figure 4b shows that cathepsin D and LAMP1 were localized in the lysosome in the MCF-7 and HepG2 cell lines through the yellow spots in the overlapped images.

### Evaluation of ER stress

**Fig. 5:**
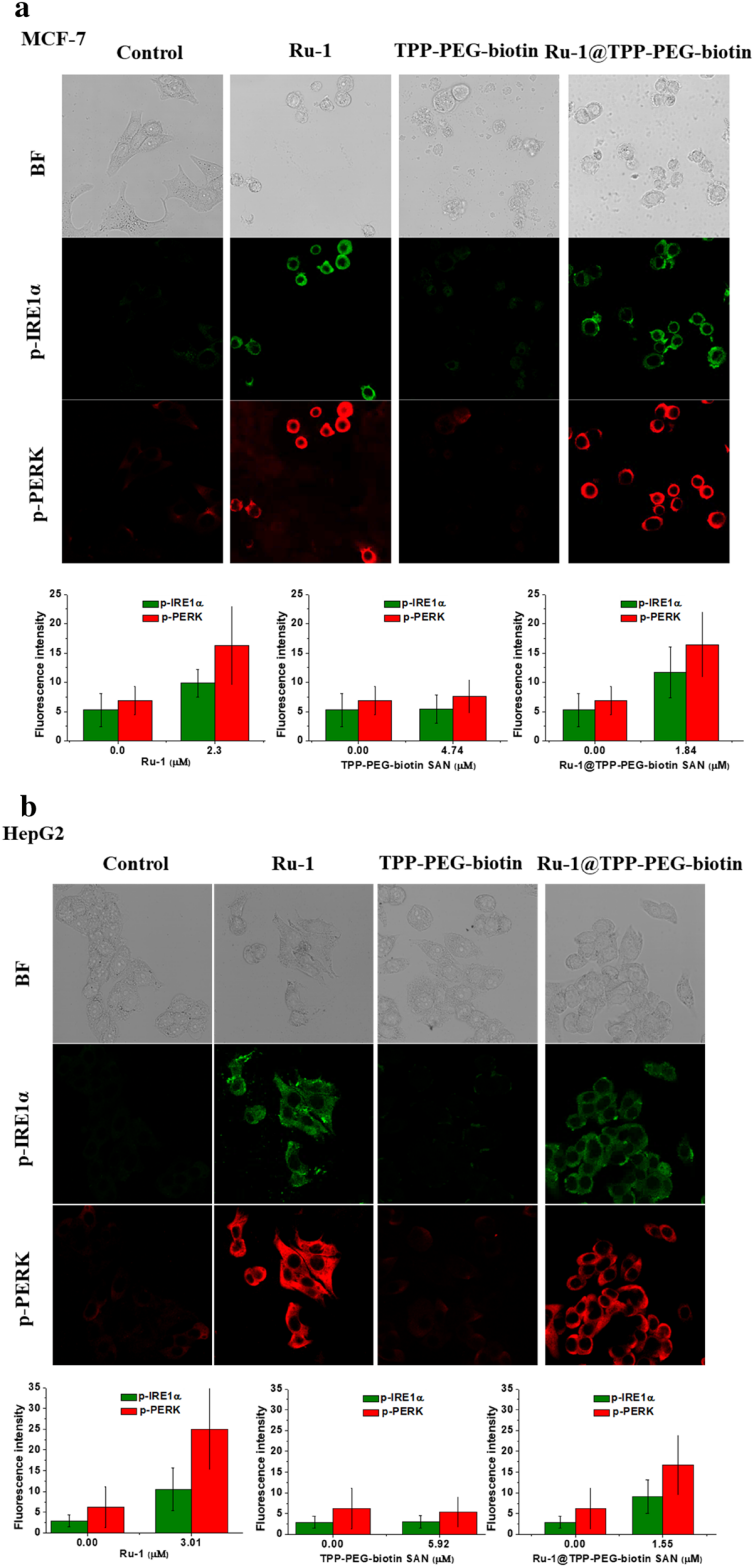
Evaluation of ER stress. The ER stress caused by Ru-1 in the MCF-7 and HepG2 cells was observed using confocal fluorescence microscopy. The confocal fluorescence microscopic images of the MCF-7 (**a**) and HepG2 cells (**b**) were obtained after the treatment with Ru-1, TPP-PEG-biotin SANs, and Ru-1@TPP-PEG-biotin SANs at their IC_50_ concentrations. The bar graphs represent the average fluorescence intensities of p-IRE1α and p-PERK in the confocal microscopic images of the MCF-7 and HepG2 cells

The unfolded protein response (UPR) is activated through ER stress caused by accumulating unfolded or misfolded proteins in the ER. GRP78 should work to produce structurally active proteins from unfolded proteins in the ER. The Ru-1 compound can inhibit the activity of GRP78 by its binding to GRP78. After the MCF-7 and HepG2 cell lines were treated with Ru-1, the UPR signaling was investigated. The UPR is initiated through phosphorylation of ER membrane protein kinases. The PERK, IRE1α, and activating transcription factor 6 (ATF6) kinases are known to be as UPR arms to activate UPR signaling. In this experiment, the phosphorylation degree of PERK and IRE1α was observed to confirm whether ER stress could be induced by Ru-1. The MCF-7 and HepG2 cells were treated with the Ru-1, TPP-PEG-biotin SANs, and Ru-1@TPP-PEG-biotin SANs. Figure 5a and b show the fluorescence cellular images acquired using the immunofluorescence assay to evaluate the phosphorylation degree of PERK and IRE1α. The p-IRE1α and p-PERK fluorescence intensities were very weak in the control. However, the Ru-1 treatment caused the p-IRE1α and p-PERK fluorescence intensities in the MCF-7 and HepG2 cell lines to be very strong. This result signifies the UPR signaling can be activated via ER stress induced by Ru-1 that inhibits the GRP78 activity. The PDT action by the TPP-PEG-biotin SANs did not show any increased fluorescence intensities by the p-IRE1α and p-PERK. This means that TPP-PEG-biotin SANs are not involved in the induction of ER stress because they did not inhibit the activity of GPR78. The Ru-1@TPP-PEG-biotin SANs treatment led to the activation of the UPR signaling in the MCF-7 and HepG2 cell lines, due to the encapsulated Ru-1 in the TPP-PEG-biotin SANs. These results clearly show the targeting effect of Ru-1 on the inhibition of GRP78.

### Evaluation of lysosome breakdown

**Fig. 6:**
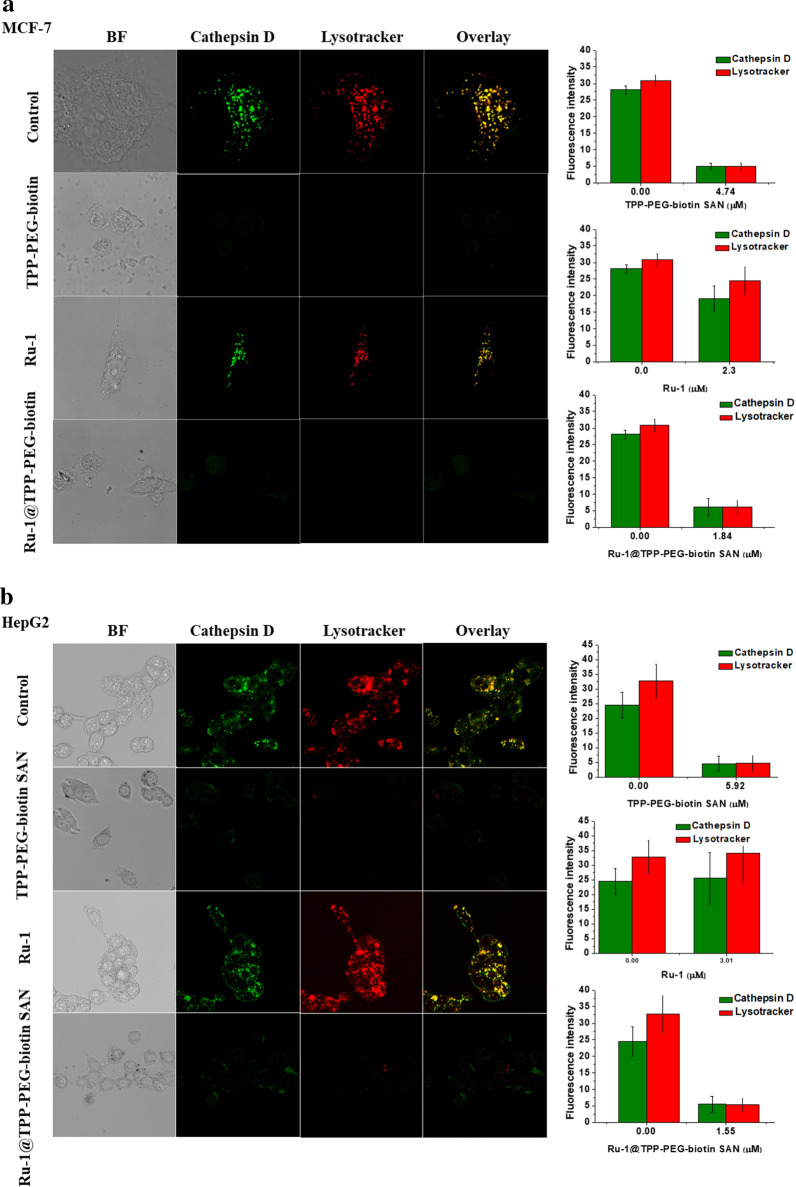

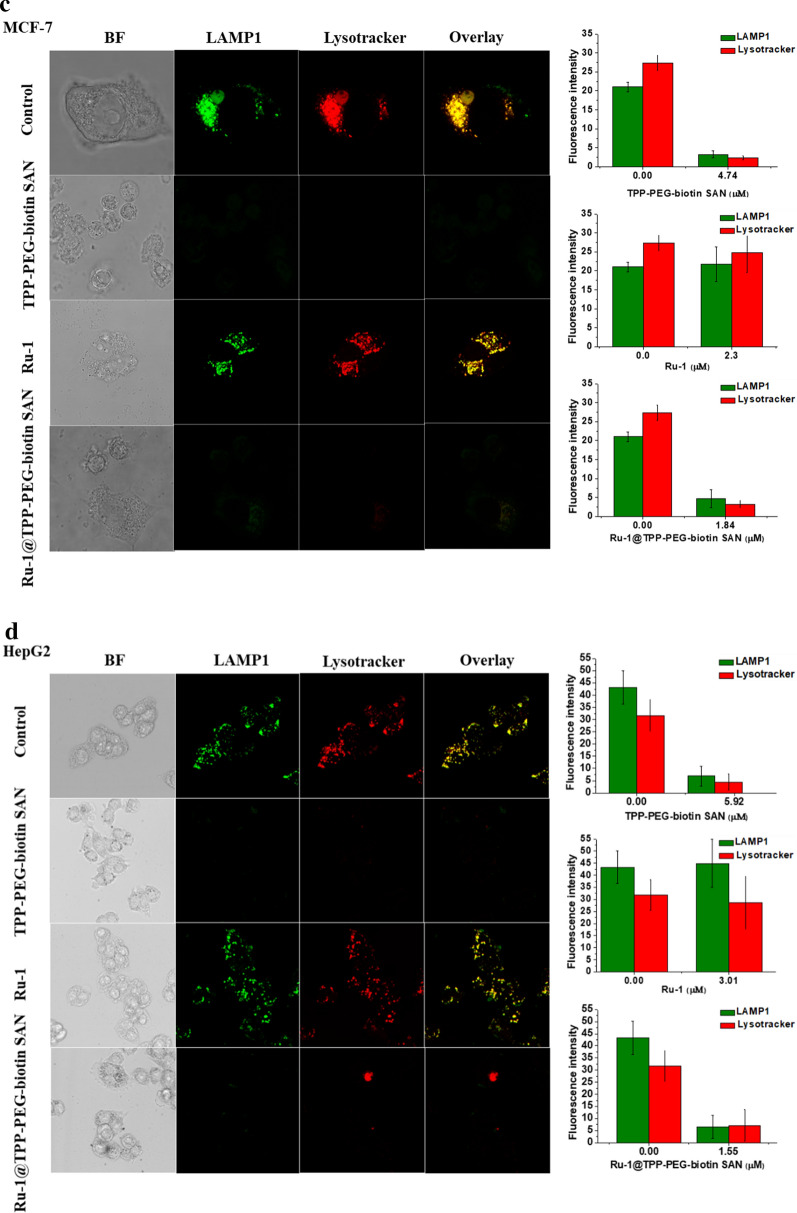
Intracellular lysosomal breakdown based on the monitoring of cathepsin D and LAMP1 in the lysosome. Lysosomal damage by the TPP-PEG-biotin SANs was observed in the MCF-7 (**a** and **c**) and HepG2 cells (**b** and **d**). The confocal fluorescence microscopic images of the MCF-7 cells were acquired after the treatment with Ru-1, TPP-PEG-biotin SANs, and Ru-1@TPP-PEG-biotin SANs at their IC_50_ concentrations. Bar graphs represent the average fluorescence intensities of the cathepsin D, LAMP1, and Lysotracker in the MCF-7 cell line

In living cells, the lysosome maintains an acidic environment. The lysotracker works as a fluorescent probe under acidic conditions such as in the lysosome. Figure 6 shows whether the lysosome is damaged by the treated therapeutic agents. Particularly, the TPP-PEG-biotin SANs as a photosensitizer and the NDDS encapsulating Ru-1 should be active to damage the lysosome because it is localized in the lysosome. Along with the lysosome stained with the lysotracker, cathepsin D and lysosomal-associated membrane protein 1 (LAMP1) were monitored. Cathepsin D and LAMP1 are localized in the lysosome as long as the lysosome is not damaged by the treated therapeutic agents. The MCF-7 and HepG2 cell lines had identical responses to the therapeutic agents. As shown in Fig. 6a and b, cathepsin D was found to be colocalized in the lysosome when the MCF-7 and HepG2 cell lines were treated with Ru-1. This indicates that Ru-1 does not cause serious damage to the lysosome. On the other hand, as a result of the TPP-PEG-biotin SANs PDT treatment, both cathepsin D and lysotracker did not give a clear fluorescence intensity. Disappearance of the lysotracker fluorescence reveals serious damage to the lysosome by the PDT action of the TPP-PEG-biotin SANs. Disappearance of the cathepsin D immunofluorescence is thought to be the release of cathepsin D from the damaged lysosome, which is, as a result, scattered into the cytosol. The Ru-1@TPP-PEG-biotin SANs treatment showed a similar tendency to only the TPP-PEG-biotin SAN treatment. As shown in Fig. 6c and d, the treated Ru-1, TPP-PEG-biotin SANs, and Ru-1@TPP-PEG-biotin SANs had similar responses with respect to LAMP1 in the lysosome like cathepsin D in the lysosome. From Fig. 6, it was verified that the lysosome was destroyed by the TPP-PEG-biotin SANs and Ru-1@TPP-PEG-biotin SANs due to their PDT action.

### GRP78 targeting and lysosomal ceramide expression

**Fig. 7:**
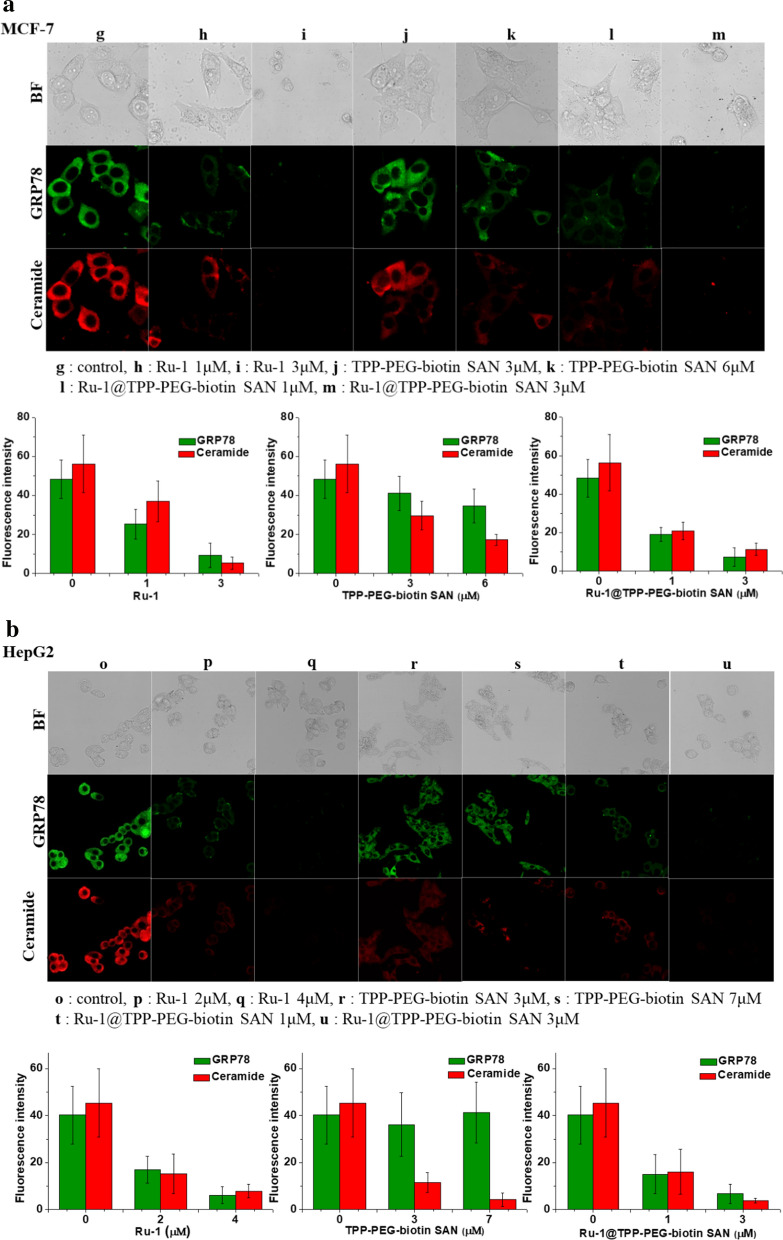
Intracellular GRP78 and lysosomal ceramide expression. Expressions of GRP78 and ceramide in the MCF-7 (**a**) and HepG2 cell line (**b**) were monitored after the treatment with Ru-1, TPP-PEG-biotin SANs, and Ru-1@TPP-PEG-biotin SANs as a function of the concentration. Bar graphs represent the average fluorescence intensities of GRP78 and ceramide in the MCF-7 and HepG2 cell lines

GRP78 is known to influence the stability of lysosomal membrane through the formation of lysosomal ceramide by GRP78-mediated activation of acid sphingomyelinase (ASM) [25]. Figure 7 shows the efficacy of the GRP78 inhibition by the tested therapeutic agents and the resultant inhibition of ceramide generation in the lysosome. As revealed in Fig. 7a, Ru-1 directly inhibited GRP78 in the MCF-7 cell line. Ru-1 can produce ROS, which causes structural changes and the dissociation of proteins. Therefore, Ru-1 localized in the ER can lead to degradation of GRP78 mainly localized in ER. As a result of the Ru-1 treatment, the immunofluorescence of GRP78 disappeared in Fig. 7a. In addition, it was found that ceramide expression was reduced to an extent in which the ceramide immunofluorescence was hard to monitor. This result can be thought to be caused by the inactivated GRP78-mediated inhibition of lysosomal ceramide production. In the case of the TPP-PEG-biotin SAN PDT treatment, the GRP78 immunofluorescence did not change. On the other hand, the immunofluorescence intensity of ceramide was greatly reduced due to the ROS-mediated degradation of the lysosome by the TPP-PEG-biotin SANs. The Ru-1@TPP-PEG-biotin SAN treatment reduced the immunofluorescence intensities of both GRP78 and ceramide. It can be deduced that both Ru-1 and TPP-PEG-biotin SANs contributed to the inhibition of the lysosomal ceramide. This result clearly demonstrates the co-targeting efficacy of GRP78 and lysosome by the Ru-1@TPP-PEG-biotin SAN combination therapy. This tendency was observed similarly in the HepG2 cell line treated with the therapeutic agents shown in Fig. 7b.

## Discussion

Traditional anticancer therapies, such as non-targeted and single drugs, are being slowly discontinued in clinical trials. Combination therapy has become a future replacement in cancer treatment because combination therapy has synergistic effects and minimize side effects. Combination therapy based on chemo drugs has been achieved by different methods. For example, co-delivery of multiple chemo drugs [26, 27], antibody–drug conjugates (ADC) [28], small molecule drug conjugates (SMDCs) [29], and combination of photosensitizer with chemo drugs [18, 30, 31] for targeted cancer therapy have been developed. The combination of two chemo drugs such as docetaxel and prednisone was the first treatment for prostate cancer (PCA). After that, several combination chemo-therapies have been developed for PCA. Recently, Li et al. developed the combination chemotherapy for PCA, such as docetaxel and doxorubicin co-delivery nanoparticles through a self-assembly process by using hyaluronic acid (HA) and cationic amphipathic starch [27]. Sometimes hematotoxicity was often observed in combination chemotherapy. Beyond that, many combination chemo-therapies have been applied to various cancer treatments. The ADC technology is another approach for targeted combination therapy, which links potent anticancer drugs with cancer cell targeting monoclonal antibodies (mAbs). Trial et al. developed the first generation antibody (BR96 mAb) conjugated chemo drug ADC therapy [32]. Even though the preclinical data were promising, the BR96-DOX conjugate failed in human trials due to gastrointestinal toxicities. Compared with the ADC therapy, SMDCs provided a new aspect for the targeted delivery of cancer drugs because it has properties such as non-immunogenic in nature, convenient synthetic method, and low molecular weights. Recently, Wang et al. reported on targeted combination chemotherapy based on SMDCs for the treatment of breast and colon cancers [33]. They designed and prepared the SMDCs using the chemo drug paclitaxel with degarelix via disulfide bonds. The in vitro assay results show that the SMDC conjugates were more cytotoxic in the MCF-7 breast cancer and HT-29 colon cancer cells than in normal cells.

Nowadays, chemo-PDT models containing a PEG moiety in the drug delivery vehicle have also been developed for combination therapy [30]. PEG has been widely used in drug delivery applications due to its biocompatibility, nontoxicity, and water solubility [34]. Without hydrophilic drug delivery polymers such as PEG and the targeting moiety may have further undesired side effects. Recently, Saravanakumar et al. prepared amphiphilic micelles for combination PDT-chemotherapy [35]. They encapsulated the PS chlorin e6 and chemo drug DOX into copolymer micelles composed of hydrophilic PEG and hydrophobic polycaprolactone (PCL). The hydrophilic polymer PEG can be covalently conjugated to PS. For example, Chung et al. reported on PEG-conjugated Gold(III) porphyrin SANs as a drug carrier and loaded the chemo drug DOX into it for a combination chemotherapy [36]. Zhang et al. developed carrier-free SANs via co-assembly of DOX and Ce6 for combination therapy [37]. They prepared DOX/Ce6 SANs using the electrostatic, π–π stacking, and hydrophobic interactions between the DOX and Ce6 molecules in an aqueous solution. In the present study, the TPP-PEG-biotin SAN contains a PEG moiety contributing to a long circulation and less side effects, and had a PS role as a therapeutic agent. Because TPP-PEG-biotin can work as a PS anticancer agent and NDDS, a co-target therapeutic approach could be attempted by encapsulating a chemo drug using TPP-PEG-biotin. The TPP-PEG-biotin has excellent multifunctional activities that simultaneously covers the PS and NDDS and targeting to cancer cell with help of the biotin moiety. This could be achieved because TPP-PEG-Biotin was synthesized through the covalent bonding of three different functional moieties. As an encapsulated chemo drug in the self-assembled TPP-PEG-biotin NDDSs, the Ru-1 complex was selected because it showed an excellent anticancer activity against drug resistant cancer cells (MCF-7 and HCT-116) and targeted GRP78 successfully.

Another important aspect of combination therapy is the drug target. A variety of targets such as DNA, cellular lipid membranes, membrane proteins, enzymes, and cytosol proteins have been tested by combination therapy [38]. Recently, subcellular organelles have received great attention as targets [39]. In this study, for the first time, the co-targeting of GRP78 and lysosome as a subcellular organelle was attempted by Ru-1 loaded TPP-PEG-biotin NDDSs. The anticancer activity of PS used in this study is based on producing ROS that have a very short lifetime in the nanosecond range. As a result, the subcellular organelle where the PS is localized becomes a drug target. The lysosome was found to be a drug target of TPP-PEG-biotin in this study. Lysosome has an important role in removing dysfunctional subcellular organelles and proteins. On the other hand, the Ru-1 compound mostly distributed in the ER suppresses the elevated levels of GRP78 which makes unfolded proteins structurally active proteins.

When the MCF-7 and HepG2 cell lines were treated with Ru-1, ER stress was generated due to the inhibition of GRP78 by Ru-1 producing ROS. This was verified through the activated UPR signaling by the phosphorylation of PERK and IRE1α, which are transmembrane proteins of the ER, shown in Fig. 5. Under the condition of non-stress, GRP78 combines with PERK, IRE1α, and ATF-6 to maintain them in a non-phosphorylated condition. GRP78 can be separated from PERK, IRE1α, and ATF-6 when ER stress occurs and UPR signaling initiates. Another noticeable aspect of GRP78 is that it is involved in the stabilization of the lysosomal membrane [40]. It is known that the lack of GRP78 increases the instability of the lysosome membrane. GRP78 is moved into the lysosome via the late endosome and interacts with BMP to act as a cofactor for the ASM enzyme. Consequently, the activated ASM by the association with BMP dissociates the lipid sphingomyelin to ceramide, which is known to be responsible for the stabilization of the lysosomal membrane. As shown in Fig. 7, the GRP78-mediated stabilization of the lysosomal membrane could be inhibited by the Ru-1 treatment. The dissociation to the ceramide was reduced such that it was hardly observed in the MCF-7 and HepG2 cell lines after the Ru-1 treatment. It was thought that the noticeably reduced ceramide contributed to the instability of the lysosomal membrane, which made the lysosome more fragile.

Because GRP78 has to produce structurally active proteins from unfolded proteins, the inhibition of GRP78 by Ru-1 unavoidably leads to the accumulation of unfolded proteins, which is a reason for the ER stress. Metabolic stress acts as a potent stimulant in the aging and apoptosis of cancer cells. Degenhardt et al. reported that cancer cells in the metabolic stress condition reduced stress, delayed apoptosis, and increased the survival of cancer cells by their autophagy action [41]. On the other hand, when the stress was not removed, cell cycle was stopped, and gene expression was adjusted so that the cancer cells could not survive. Autophagy as a defense against ER stress has an essential role in cell survival through digesting intracellular constituents in nutritional deficiencies or stress environments [42]. Autophagy-induced digesting leads to enhancing the energy efficiency and supplying raw materials for protein synthesis by breaking down dysfunctional subcellular organelles and proteins. The autophagy process is known to occur via autophagosomes isolating unfolded proteins or dysfunctional subcellular organelles, and subsequently, autophagolysosomes are formed by the fusion of autophagosomes and lysosomes containing hydrolytic enzymes to decompose unfolded proteins and subcellular organelles [43]. Autophagy degradation of by-products promotes the tricarboxylic acid cycle (TCA cycle) and production of adenosine triphosphate (ATP) by the mitochondria [44]. The resultant ATP generation helps cancer cells overcome the Ru-1-induced ER stress and contributes to promoting survival. The possibility of autophagy-mediated cancer cell survival can be destroyed as long as the lysosome malfunctions. Direct target of the lysosome by TPP-PEG-biotin should cause much more damage to the lysosome in addition to GRP-78 inhibition-induced lysosomal instability. The direct destructive approach against autophagy-mediated cancer cell survival is a fundamental key idea to attempt the co-targeting of GRP78 and the lysosome. This is why TPP-PEG-biotin was selected as the therapeutic agent to target the lysosome, along with Ru-1 to inhibit the activity of GRP78. The degradation of the lysosome leads to the release of lysosomal proteasomes such as cathepsin D into the cytosol. As a result, autophagy degradation by-products are hard to produce. The TPP-PEG-biotin-mediated degradation of the lysosome was clearly confirmed by the disappearance of cathepsin D and LAMP1 in the lysosome after the treatment with the TPP-PEG-Biotin. Considering the above results, the present co-targeting strategy is thought to be very effective as a new therapeutic approach to induce ER stress by the inhibition of GRP78 and to prevent autophagy-mediated cancer survival through the degradation of the lysosome by TPP-PEG-biotin. This was quantitatively confirmed through the IC_50_ value reduced by the co-targeting.

## Conclusions

This study showed a new anti-cancer treatment strategy based on the co-targeting of GRP78 and the lysosome. Ru-1@TPP-PEG-biotin SANs inhibited GRP78 to induce ER stress and contributed to the instability of the lysosomal membrane through the reduced expression of ceramide. Furthermore, it could break down the lysosome through the PDT action of PS localized in the lysosome which participated in the autophagy process for cancer cell survival. The co-targeting effect using the Ru-1@TPP-PEG-biotin SANs was monitored clearly by the reduced IC_50_ value compared to the TPP-PEG-biotin or Ru-1 alone.

## Materials and methods

### Materials

All chemicals and reagents purchased from sigma Aldrich and Daejung chemicals South Korea, and TCI chemicals, Japan without further purifications. Absorption spectra and drug release study were recorded in 1 cm quartz cuvettes using Evolution™ 60 UV–visible Spectrophotometer (Thermo Fischer Scientific, USA). Emission spectra were obtained on a Jasco-FP 6500 spectrofluorometer. Particle size was analyzed by Dynamic Light Scattering Spectrophotometer (Otsuka Electronics Co., Ltd, Japan). Zeta potential was measured by electrophoretic light scattering (ELS) spectrometer (Otsuka Electronics Co., Ltd, Japan). The morphology of the Ru-1@TPP-PEG-Biotin SAN was analyzed using Energy-Filtering Transmission Electron Microscope (EF-TEM, Carl Zeiss, LIBRA 120, Germany). Fourier transform infrared (FTIR) spectroscopy experiments performed using a JASCO, FT/IR-4200 instrument. The human breast cancer cell line MCF-7 and hepatoma cell line HepG2 were supplied from the Korean Cell Line Bank (KCLB, Korea). Quantum dot conjugation kit (Invitrogen, USA) was used to conjugate the antibody with a quantum dot.

### Preparation of Ru-1 and TPP-PEG-biotin

The detailed synthetic scheme and preparation methods of Ru-1 and TPP-PEG-biotin were reported previously [11, 12]. Briefly, first, the TPP-PEG-biotin was synthesized by covalently conjugating aminated-TPP (TPP-NH_2_) and carboxylic acid functionalized PEG conjugated biotin (HOOC-PEG-biotin) through EDC coupling chemistry. The triazine core functionalized ruthenium metal complex (Ru-1) was synthesized by refluxing an equivalent molar ratio of the ligand bdpta (4-(4,6-bis(3,5-dimethyl-1H-pyrazole-1-yl)-1,3,5,-triazine-2-yl)-*N*,*N*-diethylaniline) and terpyridine ruthenium trichloride ([Ru(tpy)Cl_3_]) in ethylene glycol at 170 °C overnight under N_2_ protection.

### Preparation of Ru-1@TPP-PEG-biotin self-assembled nanoparticles

The Ru-1@TPP-PEG-biotin SANs were prepared according to the previously reported procedure [12]. Briefly, 1 mmol of TPP-PEG-biotin was dissolved completely in dichloromethane (5 ml), and then, the dichloromethane was dried under a mild flow of nitrogen gas at room temperature to get a film layer. The film layer was hydrated with PBS containing 1 ml of Ru-1 (0.75 mg/ml). Subsequently, the solution mixture was sonicated for 15 min and aged for 24 h. After aging the solution for 24 h, the Ru-1@TPP-PEG-biotin SANs were finally formed. The free Ru-1 was removed by centrifugation at 15,000 rpm using an Amicon Ultra-15 centrifugal filter tube (3 kDa, Merck Millipore, South Korea). Finally, the Ru-1 loaded TPP-PEG-biotin SANs were purified by repeated centrifugation and washing with DW water, then redispersed in PBS, and stored at 4 °C for in vitro use.

### Drug encapsulation efficiency and drug loading content

The encapsulation efficiency of the Ru-1@TPP-PEG-biotin SANs was measured according to the previously reported method [12, 21]. To determine the drug loading efficiency, the Ru-1 loaded TPP-PEG-biotin SANs were prepared by sonication-assisted self-assembly. Briefly, 2 mmol of TPP-PEG-biotin and 2 mmol (1.5 mg) of Ru-1 were mixed with a DMSO/PBS (0.2 ml/0.8 ml) mixture and sonicated for 15 min. The prepared Ru-1 loaded TPP-PEG-biotin solution was incubated at room temperature for overnight. Then the Ru-1@TPP-PEG-biotin SANs was centrifuged and collected the nanoparticle. The collected Ru-1@TPP-PEG-biotin SANs was disintegrated with acetonitrile and unloaded Ru-1 was collected using an Amicon Ultra-15 centrifugal filter tube (3 kDa). Finally, the quantity of Ru-1 was measured by UV–vis absorption spectroscopy at 500 nm. The encapsulation efficiency was calculated using the following equation: Encapsulation efficiency (%) = (amount of Ru-1 agent in the NP solution/total weight of Ru-1 agent added initially) × 100. The calculated Ru-1 loading efficiency of the Ru-1@TPP-PEG-biotin SANs was 81.75%.

Drug loading content (%) = [amount of drug in NP solution/(amount of drug in nanoparticle + amount of TPP-PEG-biotin added initially)] × 100. The calculated drug loading content is 13.29%.

### Characterization of the SANs

The zeta potential and size of the particle were analyzed using dynamic light scattering and electrophoretic light scattering spectrometers. The in vitro size and charge stability SANs was performed by mixing the nanoparticle with 3 ml of 1 × PBS (pH 7.4) and incubated at 37 °C. Then, the nanoparticle solution was withdrawn at different time intervals (0, 1, 3, and 5 days) and analyzed using DLS and ELS. The morphology of the Ru-1@TPP-PEG-biotin SAN was analyzed using Energy-Filtering Transmission Electron Microscope. Briefly, 8 µl of the self-assembled nanoparticle solution in PBS was dropped on the copper grid and dried for 2 days. The size and shape of the SANs were then analyzed using TEM. The Ru-1 loaded TPP-PEG-biotin SAN was also confirmed by Fourier transform infrared (FTIR) spectroscopy using a JASCO, FT/IR-4200 instrument at room temperature. Spectra were recorded in the range of 4000–600 cm^−1^.

### In vitro release study

In vitro release study of Ru-1 loaded TPP-PEG-biotin SANs were performed by the dialysis method as reported previously [18, 21]. Dialysis was done by using Spectra/Por^®^ Dialysis membrane (molecular cut-off 12-14 KDa) against PBS as release medium. The release profiles of the Ru-1 from the Ru-1@TPP–PEG–biotin SANs were obtained using PBS at different pH at 37 °C (pH 7.4 and pH 6.0). Briefly, the sealed dialysis membrane bag containing Ru-1@TPP-PEG-biotin SANs was placed in 30 ml of PBS release medium at the specified pH at 37 °C. The release medium was agitated with shaking at 100 rpm. Then 1 ml of the released medium were taken at different time intervals (0.5, 1, 2, 4, 8, 12, and 24 h) and replaced with an equal volume of fresh PBS solution. During the release study, no precipitation was observed. The Ru-1 release was quantified by a UV–Vis spectroscopy at 500 nm. The drug release of Ru-1@TPP-PEG-biotin SANs experiments were conducted in triplicate, and the mean value of the results were detected as mean ± SD.

### Cell culture

The human breast cancer cell line MCF-7 and hepatoma cell line HepG2 were supplied from the Korean Cell Line Bank (KCLB, Korea). Cell lines were grown in Dulbecco’s modified Eagle’s medium (DMEM; Gibco, USA) supplemented with 25 μg/ml amphotericin B and 10,000 μg/ml streptomycin and 10,000 units/ml penicillin (Antibiotic–Antimycotic; Gibco, USA) and 10% (v/v) inactivated fetal bovine serum (FBS; Gibco, USA) at 37 °C in a 5% CO_2_ atmosphere.

### Intracellular drug release

The MCF-7 cells were seeded into a confocal dish (SPL Life Sciences) at a density of 2.5 × 10^4^ cells per dish in 1.0 ml of complete DMEM containing 10% (v/v) fetal bovine serum, supplemented with 1% (v/v) antibiotic antimycotic solution. After incubation for 24 h, the culture media were withdrawn and culture media containing Ru-1@TPP–PEG–biotin and free Ru-1 were supplemented. After 3 h, 12 h and 24 h, each dish was washed with 1 × PBS. For staining the nuclei, the cells were incubated with 4′,6-diamidino-2-phenylindole (DAPI) for 15 min. Stained cells were washed with 1 × PBS twice. Then, the cells were imaged using a confocal microscope (TCS SP8, Leica, Germany).

### Cytotoxicity assay

About 1.0 × 10^4^ cells were seeded into a 96-well plate and incubated overnight at 37 °C in a 5% CO_2_ atmosphere. The cells were treated with different concentrations of nanoparticles and incubated for 24 h. Then, each well was irradiated with a 660 nm diode laser at 30 mW for 20 min. After the irradiation, cells were incubated overnight at 37 °C in a 5% CO_2_ atmosphere. Subsequently, the medium containing the nanoparticles was removed, and the cells were treated with 100 μL of 0.5 mg/ml MTT solution (Thiazolyl Blue Tetrazolium Bromide; Sigma-Aldrich, USA) and incubated for 2 h at 37 °C under dark conditions. After removal of 100 μl of MTT solution, 100 μl of DMSO (Dimethyl Sulfoxide; Sigma-Aldrich, USA) were added to dissolve the formazan crystals formed by the reduction of MTT. The absorbance of the formazan solution at 540 nm was measured with a multiplate reader (Gemini XS, Molecular Devices, USA). The relative cell viability was calculated based on the measured absorbance.

### Conjugation of antibody with quantum dot

A kit (Quantum Dot Conjugation Kit; Invitrogen, USA) was used to conjugate the antibody with a quantum dot. For the conjugation of the quantum dot to the antibody, antibody carbohydrate domain modification, which is azide attachment to the antibody, and conjugation with the DIBO-modified label were performed. First, the antibodies were treated with dithiothreitol. Subsequently, the antibodies were incubated with the maleimide functionalized quantum dot. Then, the conjugations were treated with 2-mercaptoethanol to remove the maleimide group. The unconjugated quantum dots were filtered using a purification concentrator provided in the kit.

### Subcellular localization assay

The cells were plated on a confocal dish and incubated overnight at 37 °C in a 5% CO_2_ atmosphere. Subcellular localization assay was initiated when the cellular coverage reached 70%. The cells were washed with PBS and stained with the organelle-specific fluorescent probes LysoTracker and ER-Tracker (Invitrogen, USA) for 15 min in the dark. Then, the stained cells were washed with PBS, and immunofluorescence staining was done. After the staining, cells were washed with PBS, and subcellular localization of the fluorescent probes was executed using the confocal microscope (Leica microsystems, TCS SP8).

### Immunofluorescence assay

The GRP78, ceramide, cathepsin D, LAMP1, p-IRE1α and p-PERK antibodies were conjugated to quantum dots according to the manufacturer’s protocol. Approximately 1.0 X 10^5^ cells were seeded onto a confocal dish and incubated overnight at 37 °C in a 5% CO_2_ atmosphere. The cells were treated with different concentrations of nanoparticles and incubated for 24 h. Then, each well was irradiated with a 660 nm diode laser at 30 mW for 20 min. The cells were incubated overnight at 37 °C in a 5% CO_2_ atmosphere. Subsequently, the medium containing the nanoparticles was removed, and the cells were washed with PBS. Then, immunofluorescence staining was done with the antibody quantum dot conjugates. Immunofluorescence analysis was done with the confocal microscope.

## Supplementary information

**Additional file 1: Figure S1.** The intracellular Ru-1 release study of Ru-1@TPP-PEG-Biotin SANs was assessed by confocal fluorescence microscopy. (a) Confocal fluorescence images of free Ru-1 and Ru-1loaded TPP-PEG-Biotin SANs. (b) The quantitative analysis for the fluorescent intensity of Ru-1 release was measured using the software Metavue™.

## Data Availability

All data generated or analyzed during this study are included in this article.

## Abbreviations

TPP: Tetraphenylporphyrin
PEG: Polyethylene glycol
DLS: Dynamic light scattering
NDDS: Nano drug delivery system
SAN: Self-assembled nanoparticle
PS: Photosensitizer
DOX: Doxorubicin
GRP78: Glucose regulated protein 78
ER: Endoplasmic reticulum
ASM: Acid sphingomyelinase
BMP: Bis(monoacylglycero)phosphate
UPR: Unfolded protein response
PERK: Protein kinase R (PKR)-like endoplasmic reticulum kinase
IRE1α: Inositol-requiring enzyme 1 α
LAMP1: Lysosomal-associated membrane protein 1
