# Supplementary material for: Multifunctional TPP-PEG-biotin self-assembled nanoparticle drug delivery-based combination therapeutic approach for co-targeting of GRP78 and lysosome
Source: J Nanobiotechnology. 2020 Jul 20;18:102. doi: 10.1186/s12951-020-00661-y (PMC7372800; doi:10.1186/s12951-020-00661-y)
Supplement: Supplementary file 1 — Additional file 1: Figure S1. The intracellular Ru-1 release study of Ru-1@TPP-PEG-Biotin SANs was assessed by confocal fluorescence microscopy. (a) Confocal fluorescence images of free Ru-1 and Ru-1loaded TPP-PEG-Biotin SANs. (b) The quantitative analysis for the fluorescent intensity of Ru-1 release was measured using the software Metavue™. [file 12951_2020_661_MOESM1_ESM.docx]

**Additional file**

**Multifunctional TPP-PEG-biotin self-assembled nanoparticle drug delivery-based combination therapeutic approach for co-targeting of GRP78 and lysosome**

Baskaran Purushothaman, Jeongmin Lee, Sera Hong, and Joon Myong Song*

College of Pharmacy, Seoul National University, Seoul 08826, South Korea

* Corresponding author:

E-mail: jmsong@snu.ac.kr (Joon Myong Song)

**Figure S1.** Intracellular drug release


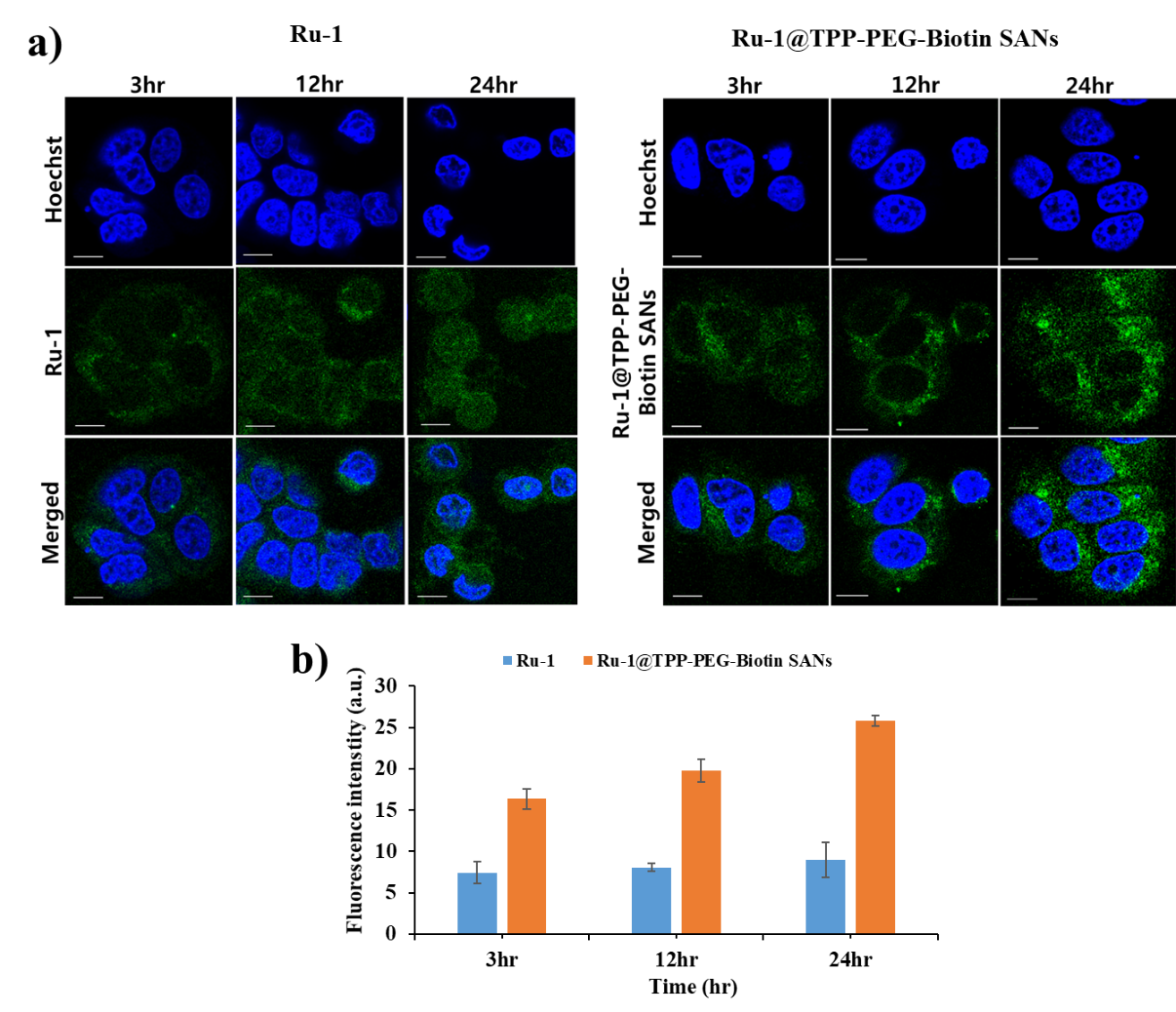


**Figure S1.** The intracellular Ru-1 release study of Ru-1@TPP-PEG-Biotin SANs was assessed by confocal fluorescence microscopy. (a) Confocal fluorescence images of free Ru-1 and Ru-1loaded TPP-PEG-Biotin SANs. (b) The quantitative analysis for the fluorescent intensity of Ru-1 release was measured using the software Metavue^TM^.
